# Technology Integration in Higher Education During COVID-19: An Assessment of Online Teaching Competencies Through Technological Pedagogical Content Knowledge Model

**DOI:** 10.3389/fpsyg.2021.736522

**Published:** 2021-08-26

**Authors:** Huma Akram, Yang Yingxiu, Ahmad Samed Al-Adwan, Ali Alkhalifah

**Affiliations:** ^1^Department of Education, Northeast Normal University, Changchun, China; ^2^Department of Electronic Business and Commerce, Al-Ahliyya Amman University, Amman, Jordan; ^3^Department of Information Technology, Qassim University, Buraidah, Saudi Arabia

**Keywords:** technology integration, online teaching practices, higher institutions, technological pedagogical content knowledge, COVID-19, teaching experience, gender difference

## Abstract

The COVID-19 pandemic significantly shifted education from traditional to an online version, which was an emergent state for teachers and students. The substantive situation thus raises the importance of technology integration in education, and teachers are required to update their competencies, respectively. In this regard, the study assessed online teaching competencies of faculty members following, technological pedagogical content knowledge (TPACK) model. Closed-ended surveys were employed for quantitative analysis of randomly selected 256 faculty members from public universities in Karachi, Pakistan. Results indicated that teachers possessed adequate levels of knowledge across all the domains of TPACK. The highest competency was obtained by content knowledge (CK), while technological knowledge (TK) was reported at the lowest level. Furthermore, a significant difference was noted in terms of gender and teaching experience. Correspondingly, the study proposes that the TPACK model should be employed in the professional development programs to develop teachers’ TPACK for integrating information communication and technology in the pedagogical practices. The findings of the study present a constructive overview of teachers’ digital competencies and technology use in teaching and learning in the time of the COVID-19 and also play a significant role in the integration of technology in the post-pandemic time in higher education. The study also suggests relevant educational authorities and policymakers for assessing and enhancing the technological competencies of teachers for quality online education.

## Introduction

As it can be seen worldwide, the COVID-19 pandemic has caused a significant interruption in all the domains of human lives. Likewise, the educational sector also encountered many challenges by the institutional closure from schools to universities, and traditional education shifted to the online paradigm (UN, 2020). The scenario of this technological transition affected the education of about half of the student population globally (UNESCO, 2020). Thereby, the situation raises the importance of technology integration in education, and teachers are required to update their competencies to endow quality education and make changes to their curriculum and instruction accordingly. Regarding the application of information communication and technology (ICT) in education, however, instructors and learners are familiar with the traditional technological teaching aids, such as Smartboards and PowerPoint; still, their practical employability is required in the teaching practices (Nikolopoulou and Gialamas, 2016; Guillén-Gámez et al., 2018). Besides, this provisional period raised the necessity, especially for the teachers, to gain competency in applying ICT in their teaching practices. Meanwhile, the application of ICT in higher education has remained a major subject of concern for decades at the global level (e.g., US Department of Education, 2017; Daniela et al., 2018). Many studies have highlighted that the application of ICT in the classroom setting has become a critical factor for meeting the needs of the learner in the knowledge society (Martins et al., 2019). Besides, the successful integration of ICT can make the learning process more exciting and keep learners motivated (Hanafi et al., 2017), which are considered as the significant predictors of their academic performance (Xu et al., 2021). In the same manner, the utilization of ICT is suggested by the government of Pakistan to optimize the educational outcomes, as it helps to enhance the pedagogical competencies of teachers and boost learners to learn actively [Pakistan Ministry of Education (MoE), 2018].

Moreover, the effective integration of ICT is essential in systematizing an efficient online educational program. The successful application of ICT not only contributes to learners’ satisfaction but also helps individuals to acquire their desired outcomes (Cervero et al., 2020). It is, therefore, essential to develop competencies in teachers to use ICT effectively in their pedagogical practices by organizing professional development programs (Guillén-Gámez et al., 2020). However, teachers’ professional training for the efficient use of ICT in teaching did not apply because of the sudden pandemic situation and put students at risk (Guillén-Gámez et al., 2020; Hong et al., 2021). Consequently, it caused excessive pressure on teachers to achieve students’ required educational attainment (Rodriguez-Segura et al., 2020). Although teachers made every effort to continue students’ learning, yet they had to encounter several challenges in adopting digital platforms for teaching, which include insufficient inter-institutional coordination (Talsma et al., 2021), little understanding, and investment in advanced technologies (Akram et al., 2021). In the past decade, however, in Pakistan, online learning has also been handled significantly, still been endured with the various constraints that prevent the effective integration of ICT in educational practices (Kanwal and Rehman, 2017; Salam et al., 2017). Earlier studies indicate that students generally show better academic performance in digital platforms comparing with the traditional ones (Shehzadi et al., 2020). On the other hand, the digital competencies of teachers are found inadequate, particularly in the formulation of lesson plans (Farid et al., 2015). However, most of the teachers are digitally literate and can conduct online lessons, yet they are found incapable of integrating ICT efficiently in their teaching practices (Al-Samarraie and Saeed, 2018). Consequently, their digital instructional approaches may remain unsuccessful in delivering the content effectively (Adnan and Anwar, 2020). In this regard, the situation raises the importance of teachers’ professional learning to acquire technological competency, as a successful pedagogical practice would mainly be possible if teachers possess a sound technological competency. The relationship between technological competency with educational content was considered necessary by Mishra and Koehler (2006) and presented this in their framework, namely, technological pedagogical content knowledge (TPACK). Their primary focus was derived on the basis of the premise that teachers are required to acquire technological competency to use it effectively in the instructional approaches. Regarding evaluation, several studies have presented instruments to evaluate the technological competencies of teachers differently, but their main focus remained on teachers’ knowledge, beliefs, and adaptation (Ertmer, 2005; Aldunate and Nussbaum, 2013; Kim et al., 2017). The complementary fact in various studies was that they comprised only one of the components of the concept.

In contrast, technological competency involves all the major components, such as knowledge (technological, pedagogical, and content), skills, and attitudes (Voogt et al., 2015), whereas limited literature and studies have been found regarding all the major components. In addition, the acquisition of TPACK depends on social, cultural, and contextual attributes, which may vary from one country to another. However, several studies have been investigated teachers’ digital competencies through all the determinants of TPACK in various countries (i.e., Lin et al., 2013; Scherer et al., 2018; Ortega-Sánchez and Gómez-Trigueros, 2019; Acikgul and Aslaner, 2020; Castéra et al., 2020). But, to the best of our knowledge, this is the first study that aims to examine teachers’ digital competencies *via* all the mentioned sub-components of TPACK during the pandemic phase, specifically in the context of higher education in Pakistan. In this regard, the present study examines the integration of ICT in faculty members’ pedagogical practices by unfolding their technological competencies level. Subsequently, lecturers and professors from public universities of Karachi city of Pakistan were considered for a case study under the guidance of the following research questions:

1. What are the levels of TPACK of faculty members across higher institutions of Karachi?
2. Is there any significant difference between faculty members’ TPACK regarding their gender and teaching experience?

## Review of Literature

### Online Teaching Competencies

The term online teaching can be exemplified with the help of these principles: (1) The learner and teacher interconnected with each other distantly *via* different digital platforms, (2) learning and learning materials can be accessed through technology, (3) the interaction between teacher and learner takes place *via* technology, and (4) teacher assists learner with the help of different digital channels of communication (Anderson, 2011a). In a general manner, online teaching is viewed as similar to the teaching for all other formal learning/teaching environments (Anderson, 2011b). On the other hand, teaching competency signifies the skills, attitudes, and knowledge of the teachers that enable them to perform in a way that meets or exceeds the expected standards successfully (Richey et al., 2011). Without having adequate competencies, it is difficult for teachers to execute and organize online instructional programs efficiently as teaching is characterized by selecting a number of tactics for a specified discourse, which may involve lesson planning or instructional and learning materials. In this regard, the previous literature finds several categories and characteristics of online teaching competencies. For instance, Thomas and Graham (2017) emphasize course design as the core component of teachers’ competencies. Bigatel et al. (2012) focused on teaching behaviors and did not focus on instructional design. Contrarily, few researchers provide a brief description of teachers’ online competencies by means of personal, social, pedagogical, and technological characteristics (Guasch et al., 2010; Baran et al., 2011; Palloff and Pratt, 2013). Other researchers propose a framework to demonstrate the features of teaching competencies. Among those, the widely used and renowned model is considered as the TPACK model, developed by Mishra and Koehler (2006). The present study employs the TPACK model to investigate online teaching competencies.

### Technology Integration in Pedagogical Practices

Several studies draw attention to the importance of technology integration in pedagogical practices and imply that it does not facilitate only students but also the teacher in the learning process (Salam et al., 2019). Islam et al. (2019) indicate that the utilization of technology in teaching makes teacher competent in pedagogical as well as content areas in the classrooms and helps learners to learn efficiently by the use of technological tools. Several studies highlight the advantages of technology use for teachers. For instance, the study of Vongkulluksn et al. (2018) highlights that the teachers prefer to spend more time teaching in the classrooms, who are good at utilizing technology. Furthermore, the technological competencies of teachers enable them to adapt other teaching strategies and approaches easily; as a result, their performance gets enhanced.

Oliva-Córdova et al. (2021) ascertain that the usage of technology in teaching practices enables learners to learn effortlessly; however, its efficient application generally depends upon teachers’ technological and pedagogical competencies. Various studies have identified the importance of these competencies and knowledge of teachers in teaching practices. Ifinedo et al. (2020) indicate that teachers’ technological knowledge either explicitly or implicitly contributes significantly to integrating ICT successfully, while teachers’ ICT pedagogical practices have found the lowest technology integration predictor. The results further suggested including professional training to assist teachers in integrating ICT efficiently by enhancing their technological competencies. To investigate the impact of teachers’ training programs on their online teaching effectiveness, Brinkley-Etzkorn (2018) conducted a survey. The findings revealed a significant change in teaching competencies and designing course syllabi in teachers, while no significant difference in teaching was observed according to their student perceptions.

**Figure 1:**
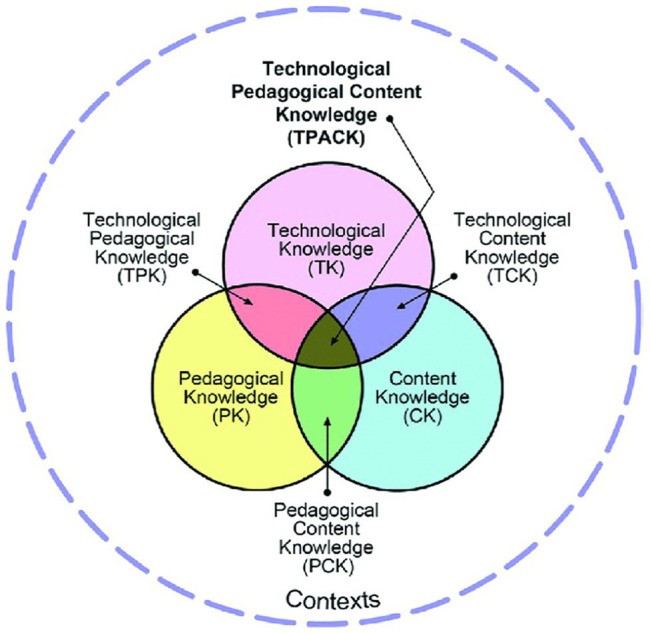
The technological pedagogical content knowledge (TPACK) framework (retrieved from http://tpack.org).

Moreover, the knowledge of technology and expertise in the utilization of technology are considered two different modes of competencies (Instefjord and Munthe, 2017). For instance, it is identified by some studies that despite having technology literacy, teachers were not capable of using technology in teaching efficiently (Dinçer, 2018; Alanazy and Alrusaiyes, 2021). It indicates that technological knowledge and using technology in pedagogical practices are two different concepts. Several studies and theories have been proposed to highlight this aspect. Briefly, it can be summarized that the effective use of technology in teaching practices is possible only if teachers are equipped with all the fundamental competencies (Ifinedo et al., 2020). Likewise, the TPACK model also ascertains that ICT cannot be integrated efficiently in educational practices until teachers do not possess all the essential technological skills (Mishra and Koehler, 2006). This model is comprised of three main components of teachers’ knowledge or acquaintance (shown in Figure 1), i.e., technological, pedagogical, and content. Although all three components of the model seem different and separate knowledge domains, interfaces and associations among these core concepts establish the central point of the overall framework (Archambault and Barnett, 2010). After following the convergence of the mentioned components, knowledge of teachers can be classified as technological content knowledge (TCK), pedagogical content knowledge (PCK), and technological pedagogical knowledge (TPK), while the complete form of all components of knowledge is represented as TPACK (Schmidt et al., 2009).

### Teachers’ TPACK Concerning Their Gender and Teaching Experience

It is indicated by several empirical studies that teachers’ characteristics also play a significant role in integrating ICT, which may vary across the countries; for instance, some studies have identified a significant difference in gender with a more inclination of males toward digital instructional development than females (Marín-Díaz et al., 2020). In terms of TPACK, studies also indicate the gender difference; for instance, Lin et al. (2013) identified higher pedagogical knowledge in female teachers with lower technological knowledge. Scherer et al. (2017) revealed that in all technological domains of TPACK, male teachers reported significantly higher competencies than females. In contrast, the TCK of female teachers was reported higher than the male teachers (Ortega-Sánchez and Gómez-Trigueros, 2019). However, a study by Castéra et al. (2020) came across different results and found no significant difference between genders in terms of teachers’ TPACK.

Another element of central concern in the acquisition of digital competencies is the teaching experience of teachers. Regarding years of teaching experience, studies show mixed results. For instance, Koh et al. (2014) identified a significant difference in ICT integration concerning the teaching experience and determined that TPACK of novice teachers was higher than experienced teachers. In contrast, Jang and Tsai (2012) identified that senior teachers’ technological skills were higher than novice teachers. Therefore, the hypotheses of the study can be posited as:

H1: *“There is a significant difference between faculty members’ TPACK with respect to their gender.”*

H2: *“There is a significant difference between faculty members’ TPACK with respect to their teaching experience.”*

## Methodology

For examining faculty members’ TPACK, a quantitative survey design was employed as it was considered the most appropriate approach to gain accurate reflection *via* numerical representation (Watson, 2015). Subsequently, the study was guided by questionnaires, which were mailed and also emailed by the researcher to various universities.

### Participants of the Study

The population of the study comprises all the faculty members from public universities of Karachi, which consists of 11 universities with estimated 785 faculty members [Higher Education commission (HEC), 2015]. For ensuring a stable data analysis, the sample size was calculated by applying the Yamane Taro sample formula for a finite population (Israel, 1992) and obtained a sample size of 260 respondents. The sample size for conducting this study was appropriate, as the size of the sample between 30 and 500 at a 5% confidence level is identified as adequate (Altunışık et al., 2004). Subsequently, the questionnaires were distributed to lecturers/professors who were selected randomly from different public universities in Karachi. After excluding questionnaires with incomplete information, 256 questionnaires were considered for the data analysis. The ages of the respondents ranged from 29 to 54years, encompassing 44.1% (*n*=113) were females and 55.8% (*n*=143) were male faculty members. Their further details are presented in Table 1.

**Table 1:** Demographic statistics of the respondents.

Category	*N*	%
**Gender**		
Male	143	55.8
Female	113	44.1
**Departments**		
Social sciences	99	38.6
Natural sciences	95	37.1
Arts and humanities	62	24.2
**Age**		
20–29	44	17.1
30–39	126	49.2
40–49	86	33.5
**Teaching experience**		
Up to 1year	38	14.8
2–5years	175	68.3
>6	43	16.7

### Ethical Concerns

In order to ensure the reliability of the findings, the study followed all the ethical concerns to conduct the study. These concerns include the granted approval from the supervisor on account of the ethical committee. The other ethical concerns include assurance of the privacy and honor of the participants of the study, and questionnaires were filled after taking their consent.

### Survey Instrument

The instrument utilized in this study was adopted from the validated scale formulated by Schmidt et al. (2009), which was devised on the basis of the TPACK theoretical framework to examine teachers’ competencies within three basic domains, i.e., pedagogies, technology, and content. The said questionnaire was intensively used by other researchers (e.g., Scherer et al., 2018; Ortega-Sánchez and Gómez-Trigueros, 2019). Before conducting data, the questionnaire was modified according to the study’s approach; for instance, the questions from the domain (content knowledge) were rephrased from a specific subject to a general subject. Furthermore, the last three qualitative questions were also excluded from the survey. The modified form of the questionnaire comprised seven dimensions of 38 items, including (1) technological knowledge (TK) 7 items, (2) content knowledge (CK) 3 items, (3) pedagogical knowledge (PK) 7 items, (4) PCK 4 items, (5) TCK 4 items, (6) TPK 5 items, and (7) TPACK 8 items. The responses of each group’s items were laid down upon a five-point Likert scale extending from “Strongly Disagree” to “Strongly Agree.”

### Confirmation of the Model Fitness

In order to increase the reliability of the findings, it is imperative to align empirical data with the theoretical framework of the study. Thereby, the fitness of all the dimensions of the TPACK model was investigated through confirmatory factor analysis as shown in Table 2. The chi-square value was less than 5 (i.e., *χ*2/*df*=4.1), which indicates the significant fitness of the model (Schermelleh-Engel et al., 2003). The other indicators were also reported significant (shown in Table 2), as their values were less than the threshold values, i.e., RMSEA≤0.06, CFI≥0.95, TLI≥0.95 (Hu and Bentler, 1999); SRMR<0.05 (Cangur and Ercan, 2015).

**Table 2:** Confirmation of the model fitness.

*χ*2	*df*	*p*	RMSEA	CFI	TLI	SRMR
1154.781	275.411	0.000	0.05	0.96	0.97	0.04

### Reliability of the Instrument

The reliability of all the constructs of TPACK was investigated through Cronbach’s alpha scale. The value of each construct was above 70% (shown in Table 3), which shows satisfactory consistency, as the collected data are reviewed as reliable if the alpha value is more than 60% (Tavakol and Dennick, 2011).

**Table 3:** Reliability evaluation.

Constructs of the questionnaire	No. of items	Alpha value
Technological knowledge (TK)	07	0.73
Content knowledge (CK)	03	0.71
Pedagogical knowledge (PK)	07	0.70
Pedagogical content knowledge (PCK)	04	0.72
Technological content knowledge (TCK)	04	0.71
Technological pedagogical knowledge (TPK)	05	0.70
TPACK	08	0.75

## Data Analysis

All the collected data were analyzed by employing various descriptive and inferential statistical tests, i.e., descriptive test (mean and standard deviation) and inferential test (*T*-test and ANOVA). Subsequently, the analysis was completed by applying the receiver operating characteristic (ROC) curve, which enabled the examination of the differences between subsamples with respect to their TPACK scores. The ROC curve is a two-dimensional graphical representation of the values of sensitivity vs. 1-specificity ranges from 0 to 1, which helps in determining the difference between different subgroups (Bradley, 1997).

### Research Question 1

Technological pedagogical content knowledge of faculty members was investigated by means of descriptive statistical tests, i.e., mean and standard deviation, which are shown in Table 4. Knowledge of all the domains of TPACK was rated above 3, which demonstrates that faculty members possess adequate knowledge as *M*≥3 (Rabe-Hesketh and Everitt, 2003). Among all domains of TPACK, the highest mean value was obtained by the content knowledge (CK), i.e., 4.6, while technological knowledge (TK) obtained the least mean value.

**Table 4:** Descriptive analysis of teachers’ TPACK.

Factors of TPACK	*M*	*SD*
Technological knowledge (TK)	3.1	0.81
Pedagogical knowledge (PK)	4.1	0.69
Content knowledge (CK)	4.6	0.21
PCK	4.2	0.65
TCK	3.4	0.79
TPK	3.3	0.74
TPACK	3.2	0.71

### Research Question 2 (Hypotheses)

Before checking hypotheses, the normality test was conducted through Shapiro–Wilk test to know whether the data meet the criteria of conducting a parametric test since it is considered the most prevailing test to investigate normality (Razali and Wah, 2011). Results showed that the data were normally distributed as S-W value was 0.83 and the significant value was greater than 0.5, i.e., 0.61, which allows parametric tests to be conducted. Subsequently, the posited hypotheses of the study were checked by employing inferential statistics, i.e., *T*-test and ANOVA, where *T*-test was employed to investigate the difference between faculty members’ TPACK with respect to their gender and ANOVA was applied to test the hypothesis regarding teaching experience of faculty members.

#### Hypothesis 1

All the components of TPACK were compared by applying the *T*-test (shown in Table 5). Results revealed a significant statistical difference between male and female respondents (i.e., *T*=10.34; *p*=0.000) at alpha level 0.05. Therefore, the hypothesis regarding faculty members’ TPACK with respect to their gender was accepted. Furthermore, male faculty members got a significantly higher mean score (4.12) than the female teaching faculty (3.67), which shows that the TPACK of male faculty members was greater than the female ones.

**Table 5:** *T*-test analysis by gender of teachers.

Gender	*N*	Mean	*SD*	*df*	*T*	Sig
Male	143	4.12	0.78	142	10.34	0.000
Female	113	3.67	0.49			

**Figure 2:**
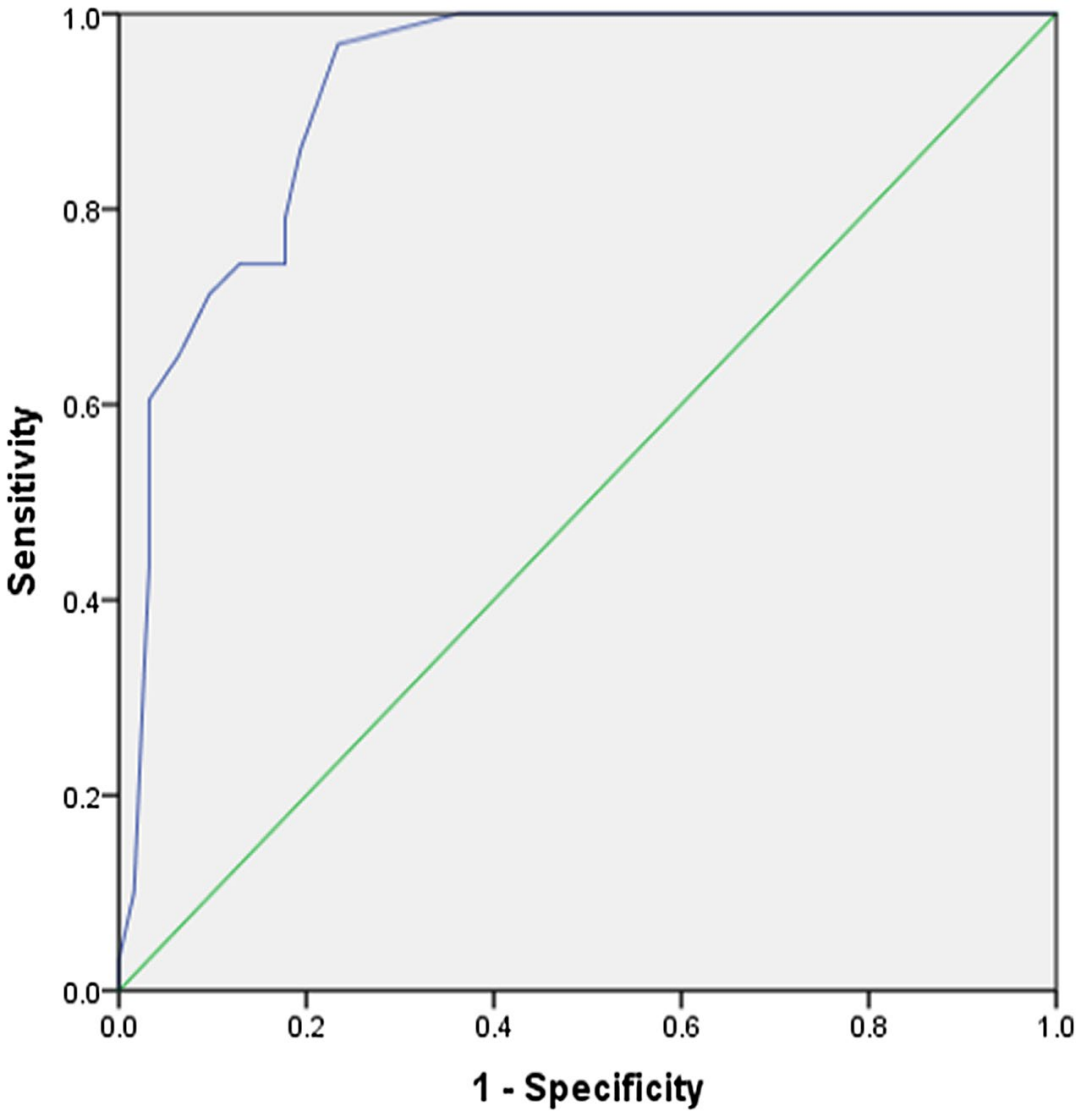
Receiver operating characteristic (ROC) curve (gender).

In addition, the gender difference with respect to TPACK scores was represented graphically through the ROC curve. The results shown in (Table 6; Figure 2) showed high sensitivity and specificity with an area under curve (AUC) of 0.921 with a significant statistical difference, i.e., *p*=0.000 at alpha level 0.05.

**Table 6:** ROC curve parameters (female gender).

AUC^a^	*CI*^b^ 95%	Standard error	Sig
0.921	0.887–0.956	0.017	0.000

a *Area under curve*

b *Confidence interval*

**Figure 3:**
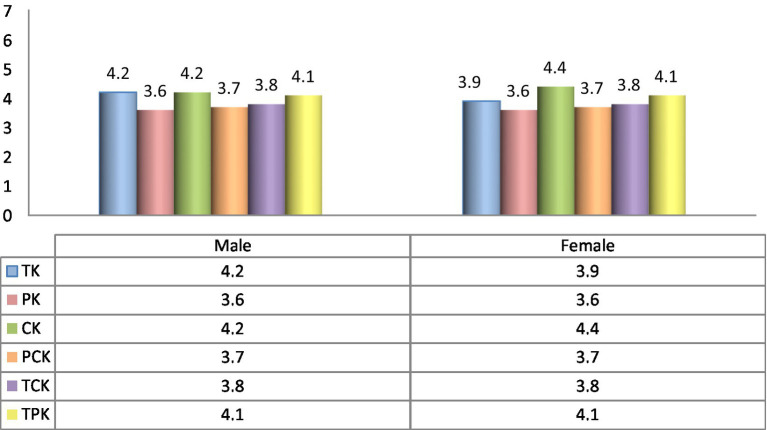
Distribution of components of TPACK by gender.

Furthermore, to investigate the most optimal predictors of teachers’ TPACK, the mean of all the sub-components was compared with respect to their gender (shown in Figure 3). Results reveal that the TK of male faculty members was significantly greater than the female ones. However, the CK was found significantly higher in female faculty members than the male ones.

#### Hypothesis 2

For examining the distinction between faculty members’ TPACK with regard to their teaching experience, the mean of TPACK was compared with the teaching experience of all the faculty members by applying the ANOVA test (shown in Table 7). Results reveal a significant difference between faculty members’ teaching experiences with their TPACK. Therefore, the hypothesis regarding the teaching experiences of faculty members was accepted, which further demonstrates that the TPACK of teachers with experience of 2–5years is higher than the novice and inexperienced teachers.

**Table 7:** ANOVA by teaching experience of teachers.

Academic interests	*N*	Mean	*SD*	*F*	Sig
Up to 1year	38	4.28	0.32	5.47^**^	0.000
2–5years	175	4.49	0.31		
>6	43	4.40	0.28		

** *p*<0.05

In order to find out the further differences across all possible pairs of the faculty members’ teaching experiences, Tukey’s honestly significant difference *post-hoc* test was conducted. Since Tukey’s HSD test helps to compare the means of all the possible pairs (Abdi and Williams, 2010). Results from Tukey’s *post-hoc* test (Table 8) demonstrate that only one out of three groups had a significant difference among each other, i.e., teaching experiences up to 1year vs. 2–5years.

**Table 8:** *Post-hoc* test.

Test	Sig
Up to 1year vs. 2–5years	0.004
Up to 1year vs. >6	0.32
2–5years vs. >6	0.446

**Figure 4:**
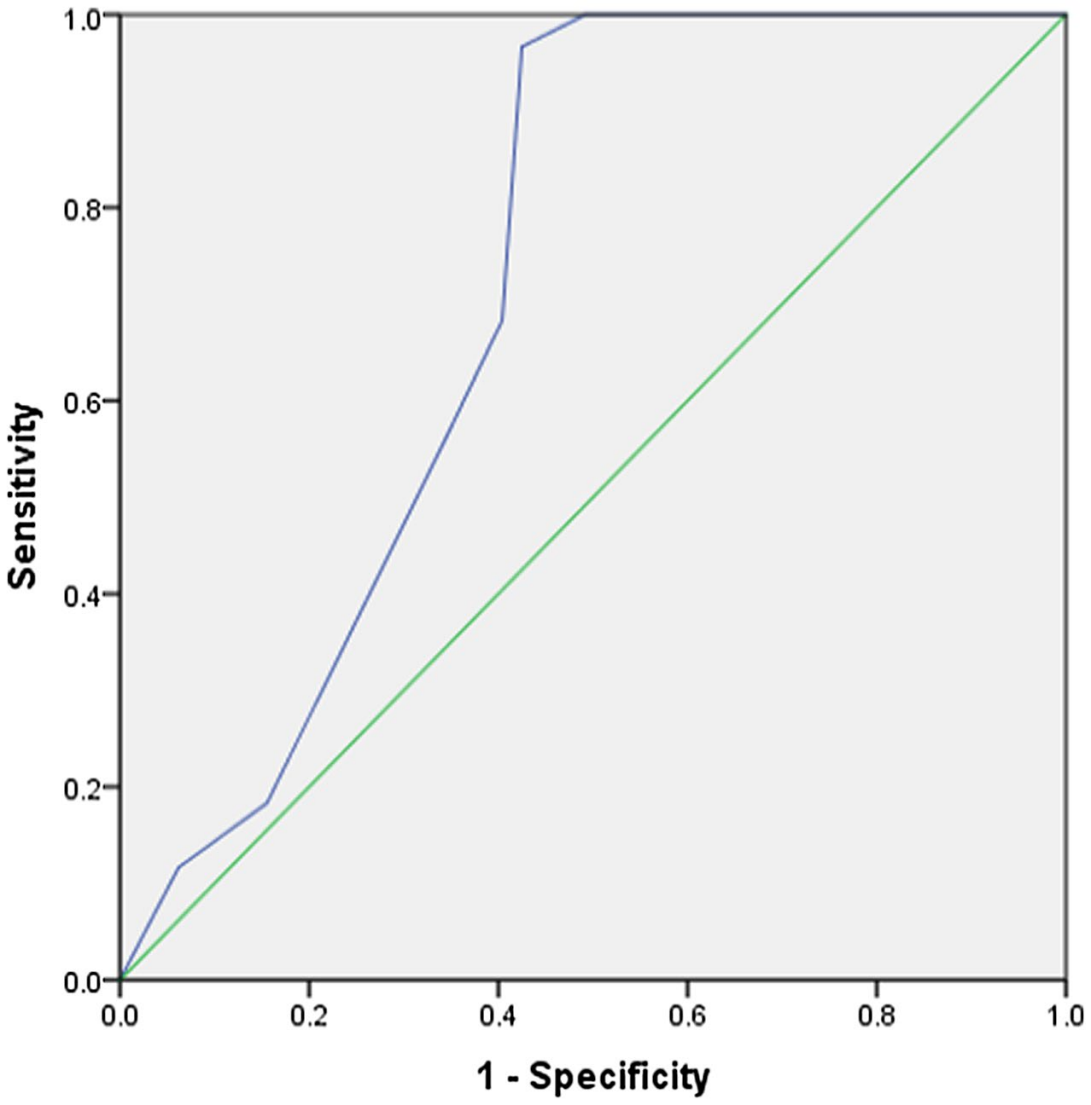
ROC curve (teaching experience).

In addition, the difference in teaching experience with respect to TPACK scores was represented graphically through the ROC curve. The results shown in (Table 9; Figure 4) illustrated high sensitivity and specificity with an AUC of 0.716 with a significant statistical difference, i.e., *p*=0.000 at alpha level 0.05.

**Table 9:** ROC curve parameters (teaching experience).

AUC^a^	*CI*^b^ 95%	Standard error	Sig
0.716	0.655–0.777	0.031	0.000

a Area under curve

b Confidence interval

## Discussion

COVID-19 outbreak has transformed the traditional educational practices and brought teaching-learning around digital format across the world. This transformation not merely raises the importance of the educational technology infrastructure of the country but also establishes a prerequisite for teachers to integrate technology in their pedagogical practices effectively to sustain students’ learning. Since the systematic implementation of ICT in teaching practices is remained at an early stage and a limited focus has been given at the higher education level. In this regard, the current study gives a deep insight to understand the levels of core competencies of faculty members’ TPACK with the role of personal variables (i.e., gender and teaching experience) in the acquisition of digital competencies during the COVID-19 period.

In view of the obtained findings, the study reveals that faculty members possess adequate knowledge in all the sub-components of the TPACK model, which shows that teachers have sufficient knowledge and skills regarding technology use in their pedagogical practices. This finding shows consistency with the findings of Mouza et al. (2014), where participants experienced a significant gain in all sub-components of TPACK. Hence, our results indicate that TPACK is an excellent framework to examine teachers’ competencies in the context of universities’ teachers of Pakistan.

The results further indicate that the highest competence of faculty members among all other domains was obtained by the content knowledge (CK), which shows that faculty members seem more confident in their content knowledge than other domains of expertise. A similar finding is also identified by Acikgul and Aslaner (2020); accordingly, teachers’ content knowledge was found adequate. In contrast, the study conducted by illustrated that teachers were confident primarily in the pedagogical knowledge (PK). It is therefore imperative to draw attention toward teachers’ content knowledge through continuous monitoring and by offering in-service workshops to sustain the students’ learning outcomes, as it helps learners to understand concerning concepts significantly.

Technological knowledge involves operating particular technologies, which plays a crucial role in integrating technology in the process of teaching and learning (Chai et al., 2010). Besides, successful e-learning can only be yielded when teachers can use technology in their pedagogical practices appropriately. On the other hand, the technological knowledge (TK) of faculty members was found at the lowest level among all other domains, which indicates that teachers lack technological competence. Thus, they require professional guidance to update their technological skills. The findings of Schmid et al. (2021) also indicated that teachers’ technological and TCK emerged as the least acquired competencies. Therefore, to enhance the technological literacy in teachers, ICT training centers with ICT professionals should be originated at the national and provincial levels.

The knowledge regarding the interaction between all domains of TPACK plays a significant role in the development of an innovative learning environment (Ortega-Sánchez and Gómez-Trigueros, 2019). However, the other reported lowest competence of faculty members was found in the domain of TPACK. This finding reflects the finding of Lye (2013), who indicated that teachers possess low TPACK, and they need improvement in several areas of TPACK. In light of this result, teachers should be given a range of guidance in terms of all the domains of TPACK and the interaction between those domains by providing both initial and ongoing support to implement the technologies in their pedagogical practices successfully.

This study further found that male teachers’ TPACK was significantly higher than female faculty members. This finding reflects the finding of Koh et al. (2010), where male teachers showed more positive attitudes, competencies, and knowledge with respect to technology use. This result indicates that female faculty requires more support to gain their competencies in all the sub-components of TPACK. Regarding teaching experience, it is usually expected that teachers’ knowledge increases with the increase in years of experience. In contrast, the results showed a statistical significance in the TPACK of faculty members’ knowledge, where faculty members with experience of 2–5years shown higher TPACK than the teachers with more experience and novice teachers. This finding supports the results of Claro et al. (2018), where years of teaching experience were found significantly associated with the TPACK of teachers. Based on the personal factors, policymakers and teachers should be aware of gender differences’ effect on technological knowledge and competencies; therefore, gender differences should be monitored closely by conducting longitudinal TPACK studies. There should be an equal emphasis on training programs on pre-service as well as in-service teachers so that teachers of all levels may learn effectively to integrate technology into their educational practices.

In addition, the study suggests that the new technological instructional context in the COVID-19 phase appeared as an important moderator for teachers in upgrading their competencies in terms of TPACK. Regarding the contextual environment, Mishra (2019) indicates that the addition of contextual knowledge (XK) may reveal the situational and institutional limitations that teachers work within. During the COVID-19 pandemic, teachers and learners experienced their practices in multiple new and unfamiliar contexts, which impacted teachers’ abilities to teach successfully remotely in the digital environment. Therefore, future studies should examine teachers’ XK comprehensively to determine the influence of different contextual factors on teachers’ TPACK with a focus on facilitating teachers with contextual change.

Finally, remote work and online learning are teaching conditions that will continue to advance steadily. In turn, the post-COVID-19 reactivation and recovery processes do not seem to contemplate that the teaching and learning processes as before. Therefore, future research should be aimed not only at understanding human behavior while studying or teaching virtually but also at understanding the TPACK model and building better ways to integrate technology into educational practices. In this regard, the findings of the study are highly significant, not particularly in determining the technology integration during the COVID-19 pandemic phase, which is currently the most crucial issue, but also for the integration of technology in the post-pandemic time in higher education.

## Conclusion

Based on the obtained results, the study affirms that the COVID-19 pandemic phase significantly influenced the digital competencies of faculty members and reveals adequate knowledge in all the sub-components of the TPACK, with a significant difference in terms of gender and teaching experience. Regarding consistency, the TPACK model was verified by means of factorial analyses in terms of seven sub-components and in the context of higher education faculty members in Pakistan, which supports the value and appropriateness of the model. Accordingly, the study suggests that the TPACK model should be employed in the professional development programs to develop teachers’ TPACK for integrating technology efficiently by bridging the gap between ICT knowledge and ICT practice.

## Implications and Limitations

The findings of this study contribute to society in several ways. Regarding theoretically, this study has enriched the literature on the technological competencies of teachers during the COVID-19 transitory period and verified the reliability of the TPACK model in the context of Karachi, Pakistan. It can be further used for verification in other cities and countries. In terms of methodological contribution, the study provides tentative insight in evaluating the impact of the COVID-19 transitory period on teachers’ digital competencies and their state of implementation in pedagogical practices. Regarding academics, this study provides a pragmatic direction to relevant educational authorities and policymakers for the improvement of online education by providing pertinent solutions and recommendations as per the situation. In addition, the future planning of professional development and training programs for the teachers can be based on the feedback provided by the faculty members. The study can further contribute to elevating e-learning outcomes and satisfaction during as well as post-pandemic phase.

Furthermore, the study also noted some limitations. Firstly, faculty’s response biasness may have affected the results since digital competencies were assessed self-reported quantitatively. Therefore, the future studies may select other approaches to unfold the understanding of teachers and establish the criteria for evaluating the TPACK of teachers. Secondly, the current study only focused on the TPACK model to assess the digital competencies of faculty. The findings of this study can be further strengthened in the future by employing other indicators to examine the teachers’ competencies in teaching with technology.

Finally, the analysis was cross-sectional and evaluated the teaching practices of university teachers during the period of the COVID-19 pandemic. Online technological, pedagogical, and content competencies of teachers may change over time, which should also be observed. Therefore, a longitudinal study should be conducted to strengthen the evidence by understanding the causal effects and interrelationships among various other variables, critical in elevating the online pedagogical practices at the higher level in Pakistan.

## Data Availability Statement

The raw data supporting the conclusions of this article will be made available by the authors, without undue reservation.

## Ethics Statement

Ethical review and approval was not required for the study on human participants in accordance with the local legislation and institutional requirements. The patients/participants provided their written informed consent to participate in this study.

## Author Contributions

HA is the principal investigator of the study. From conceptualization to the data analysis, she conducted by herself. All authors contributed to the article and approved the submitted version.

## Conflict of Interest

The authors declare that the research was conducted in the absence of any commercial or financial relationships that could be construed as a potential conflict of interest.

## Publisher’s Note

All claims expressed in this article are solely those of the authors and do not necessarily represent those of their affiliated organizations, or those of the publisher, the editors and the reviewers. Any product that may be evaluated in this article, or claim that may be made by its manufacturer, is not guaranteed or endorsed by the publisher.
